# Wing shape allometry and aerodynamics in calopterygid damselflies: a comparative approach

**DOI:** 10.1186/1471-2148-13-118

**Published:** 2013-06-07

**Authors:** David Outomuro, Dean C Adams, Frank Johansson

**Affiliations:** 1 Population and Conservation Biology, Department of Ecology and Genetics, Evolutionary Biology Centre, Uppsala University, Norbyvägen 18D, 75236, Uppsala, Sweden; 2 Department of Ecology, Evolution, and Organismal Biology, Iowa State University, 241 Bessey Hall, Ames, IA 50011, USA

**Keywords:** Geometric Morphometrics, Non-dimensional Radius of the Second Moment of Wing Area, Phylogeny, Size, Territorial Behavior, Wing Coloration

## Abstract

**Background:**

Wing size and shape have important aerodynamic implications on flight performance. We explored how wing size was related to wing shape in territorial males of 37 taxa of the damselfly family Calopterygidae. Wing coloration was also included in the analyses because it is sexually and naturally selected and has been shown to be related to wing shape. We studied wing shape using both the non-dimensional radius of the second moment of wing area (RSM) and geometric morphometrics. Lower values of the RSM result in less energetically demanding flight and wider ranges of flight speed. We also re-analyzed previously published data on other damselflies and dragonflies.

**Results:**

The RSM showed a hump-shaped relationship with wing size. However, after correcting for phylogeny using independent contrast, this pattern changed to a negative linear relationship. The basal genus of the study family, *Hetaerina*, was mainly driving that change. The obtained patterns were specific for the study family and differed from other damselflies and dragonflies. The relationship between the RSM and wing shape measured by geometric morphometrics was linear, but relatively small changes along the RSM axis can result in large changes in wing shape. Our results also showed that wing coloration may have some effect on RSM.

**Conclusions:**

We found that RSM showed a complex relationship with size in calopterygid damselflies, probably as a result of other selection pressures besides wing size *per se*. Wing coloration and specific behavior (e.g. courtship) are potential candidates for explaining the complexity. Univariate measures of wing shape such as RSM are more intuitive but lack the high resolution of other multivariate techniques such as geometric morphometrics. We suggest that the relationship between wing shape and size are taxa-specific and differ among closely-related insect groups.

## Background

Flight performance is a result of complex interactions between body morphology (i.e., wings, tails), behavior and the biological and physical environment [1-3]. In addition, body size has an important aerodynamic effect on flight performance [4,5]. One of the main reasons is that Reynolds numbers change many orders of magnitude from small to large flying animals [5]. At small body sizes, viscous effects due to small perturbations in the air are dissipated more rapidly, while at larger body sizes those small perturbations can result in stronger unsteadiness of the flow fields around wings [4]. Another aspect of morphology that has a large impact on flight performance is wing shape [6-8]. For instance, in insects, long and slender wings are optimal at long duration flight, while short and broad wings are optimal at slow and agile flight [6,7]. Moreover, a broad wing base allows a wider speed range [9] and a narrow wing tip allows less costly extensive flight [6]. Other body traits such as the centers of body and wing mass are also very important in predicting flight performance [10,11]. It is important to note that wing shape has an allometric component [12] and also a non-allometric component, since wings are complex structures and a simple proportional change of its shape with body size is unlikely [13-15]. For example, similar size clines may be achieved by different changes in wing proportions [16]. Therefore, wing shape and wing size may function as independent components of wing morphology ([13] and references therein), although wing shape would be expected to improve flight performance for a given individual size [17]. Hence, comparative studies on how wing shape changes with body size should be important for a better understanding of flight performance.

Apart from body size, other variables might also affect the optimal wing shape, for example flight behavior [2,7,18-21]. We should expect a similar wing shape within certain flight behaviors, and at the same time variation of this shape related to body size. In a comparative analysis between size and wing shape it is therefore important to take behavior into account or using species with the same behavior. Another variable that potentially could affect wing shape is the presence of ornaments on the wings that are used for sexual displays. These ornaments are typically positively sexually selected, either by male-male interactions and/or by female choice, and are condition dependent [22-25]. Wing shape may thus also play a role in ornament sexual signaling, since it might maximize both flight performance (e.g., flight style) and ornament display (e.g., size and shape of the ornament) [26-28]. For example, a large color patch at the tip of the wing should select for a broad wing tip shape [28] while a high agility (e.g., in territorial species) should select for a broad and short overall wing [6]. Hence, the optimal shape might be somewhere in between these two. Therefore, the observed wing shape would differ from that predicted by aerodynamic theory *per se*.

In this study, we explore how wing shape is related to wing size (used as a proxy of body size), and discuss its implications on flight performance. For that purpose we compare males of damselfly taxa within the family Calopterygidae. These species display territorial behavior [29]. Territorial flight is a common flight behavior in insects such as butterflies and damselflies [30,31] and is associated with broad and short wings, which are expected to improve flight agility [6,10,32]. Many calopterygid males have wing coloration that is used for signaling in sexual selection processes [23,33-39] and species discrimination [40-42], and it is also selected by bird predators [43,44]. Although calopterygid males all have more or less short and broad wings, males still show a wide variation in wing morphology, which is related to wing size and coloration [28,45,46]. The present study differs from our previous works on wing shape in Calopterygidae [28,45,46] in that here we focus explicitly on the allometric effects on wing shape and the expected consequences on flight performance.

We studied wing shape using uni- and multivariate methods. First, we quantified wing shape using the non-dimensional radius of the second moment of wing area [10,32,47] (hereafter RSM). This parameter can be defined as wing area distribution along the wing axis and is proportional to the mean lift force and hence important for energetics of flight [10,47]. Lower values involve a more basal distribution of the wing area and less energetically demanding flight with a wider range of available flight speeds [9,10]. It has also been used as an estimate of flight agility [32], although this relationship might not be as straightforward. Second, we estimated wing shape using geometric morphometrics which allowed the analysis and visualization of shape in a very precise way [48]. Since these two estimates of wing shape differ in how they capture shape and since wing shape affects flight performance, we also explored the relationship between them and with size.

In the present work, we specifically studied 37 taxa of male calopterygid damselflies that differ in wing shape, wing size and wing coloration patterns. We first explored the allometry of wing shape (using the RSM and geometric morphometrics) and the effect of wing coloration. We then compared wing shape captured by the RSM and by geometric morphometrics. We also investigated how the RSM and body length were related in a larger sample of both dragonflies and damselflies, by re-analyzing previously published data. Finally, we also performed independent contrasts for taking into account the evolutionary relationships of our study taxa.

## Results

### RSM and size

**Figure 1:**
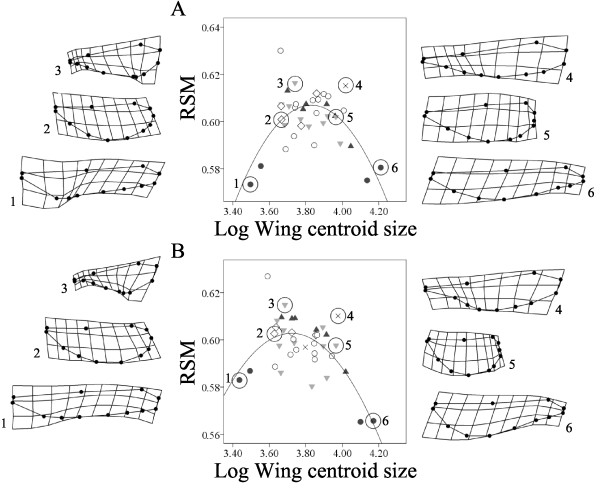
**Relationship between wing shape, measured as the non-dimensional radius of the second moment of wing area (RSM), and wing centroid size (A: forewings; B: hindwings).** Wing shape variation is shown as deformation grids obtained from geometric morphometrics. The deformation grids are based on certain taxa contained in circles and numbered. Data are grouped into the different coloration groups (see Methods; group 1: filled circles; group 2: triangles; group 3: inverted triangles; group 4: rhombuses; group 5: crosses; group 6: open circles).

Before correcting for phylogenetic effects, the RSM was related in a quadratic way to wing centroid size in both fore- and hindwings (Figure 1). Thus, the smallest and largest taxa showed the lowest values of the RSM. The quadratic term was significant both for fore- (linear regression: *R*^2^ = 0.013, *F*_1,35_ = 0.457, *P* = 0.503; quadratic regression: *R*^2^ = 0.424, *F*_2,34_ = 12.520, *P* < 0.001) and hindwings (linear regression: *R*^2^ = 0.151, *F*_1,35_ = 6.246, *P* = 0.017; quadratic regression: *R*^2^ = 0.435, *F*_2,34_ = 13.094, *P* < 0.001). Our study taxa were sorted in six different coloration groups according to the position, color and extension of wing coloration [28]. The quadratic term was mainly driven by the coloration group 1, i.e. with a wing spot located at the wing base: the genera *Hetaerina* and *Archineura* (Figure 1). Using geometric morphometrics, we visualized how the RSM was related to wing shape and wing centroid size in our study taxa. For forewings, shape changed from a long slender wing with a broad base at small size to a wing with a broad tip and narrower base at intermediate sizes, then changing towards a longer wing with a broad base and a more slender tip at larger sizes (Figure 1). Hindwings showed a similar qualitative pattern. Thus, overall wing shape did not change in a merely proportional manner with wing centroid size (Figure 1).

**Figure 2:**
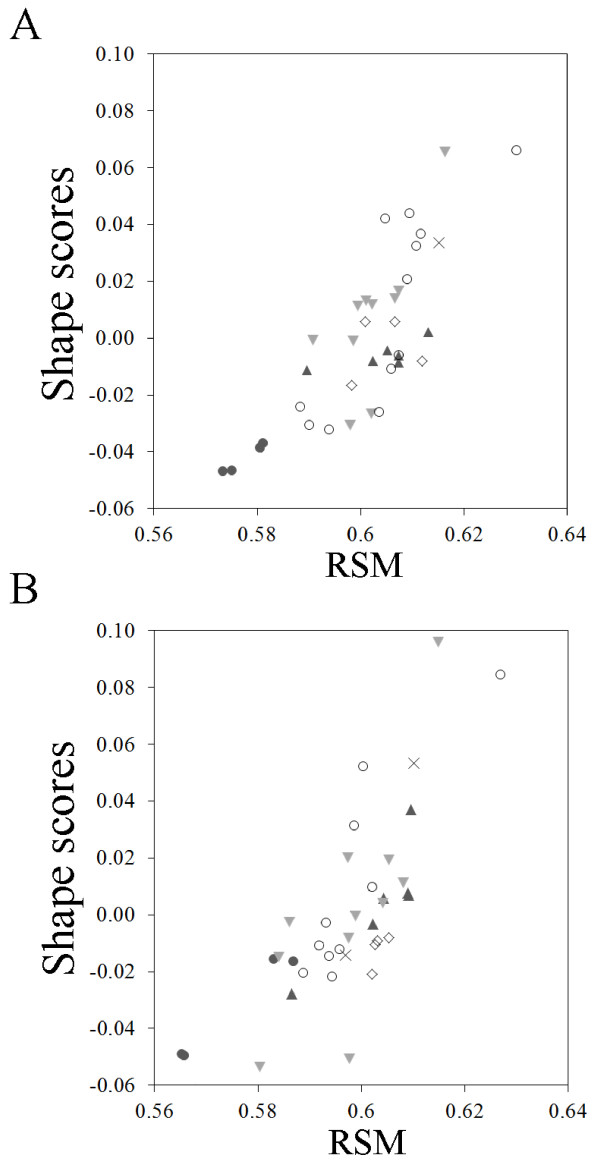
**Relationship between wing shape expressed as shape scores, and wing shape measured as the non-dimensional radius of the second moment of wing area (RSM) (A: forewings; B: hindwings).** The symbols indicate the different coloration groups (see Methods; group 1: filled circles; group 2: triangles; group 3: inverted triangles; group 4: rhombuses; group 5: crosses; group 6: open circles).

The RSM showed a linear relationship with wing shape estimated from geometric morphometric methods as shape scores, both for fore- and hindwings (Figure 2). This relationship was supported by a MANCOVA on the shape components (obtained using geometric morphometrics) (fixed factor, fore- and hindwings: Wilk’s lambda = 0.157, *F*_20,52_ = 13.984, *P* < 0.001; covariate, RSM: Wilk’s lambda = 0.035, *F*_20,52_ = 72.692, *P* < 0.001). Nevertheless, for similar values of the RSM very different shapes can be obtained, partially as a result of differences in wing size (Figures 1 and 2).

**Figure 3:**
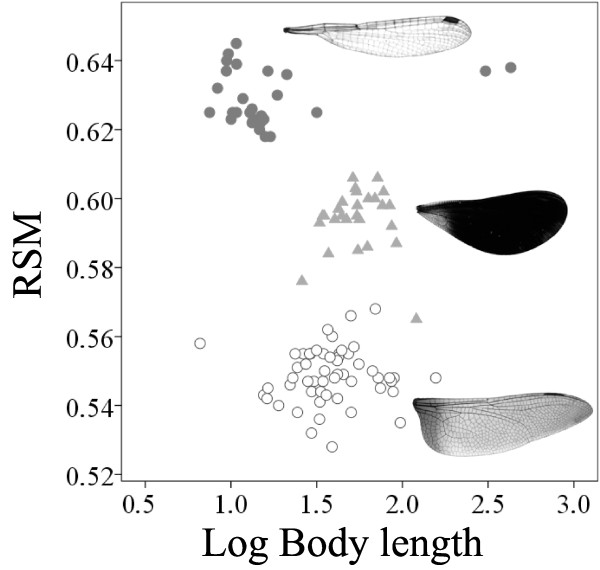
**Relationship between wing shape, expressed as the non-dimensional radius of the second moment of wing area (RSM), and body length (based on the data of Serrano-Meneses et al. **[32]**for Odonata) (dark circles: non-calopterygid damselflies; triangles: calopterygid damselflies; white circles: dragonflies).** Pictures show an example of hindwings of a non-calopterygid damselfly (*Lestes viridis*), a calopterygid damselfly (*Calopteryx maculata*) and a dragonfly (*Anax imperator*).

We also tested the relationship between the RSM and body length in a larger size range of dragonflies and damselflies, by re-analyzing previously published data [32]. The Odonata groups (calopterygid damselflies, non-calopteryid damselflies and dragonflies) differed significantly in the RSM, but body length did not show a significant effect in the model (Odonata group: *F*_2,104_ = 812.344, *P* < 0.001; Body length: *F*_1,104_ = 0.552, *P* = 0.459). Hence, we found no linear or hump-shaped relationship between the RSM and size when using a larger size range. Calopterygid damselflies showed intermediate values of the RSM with respect to non-calopterygid damselflies and dragonflies (Figure 3).

**Figure 4:**
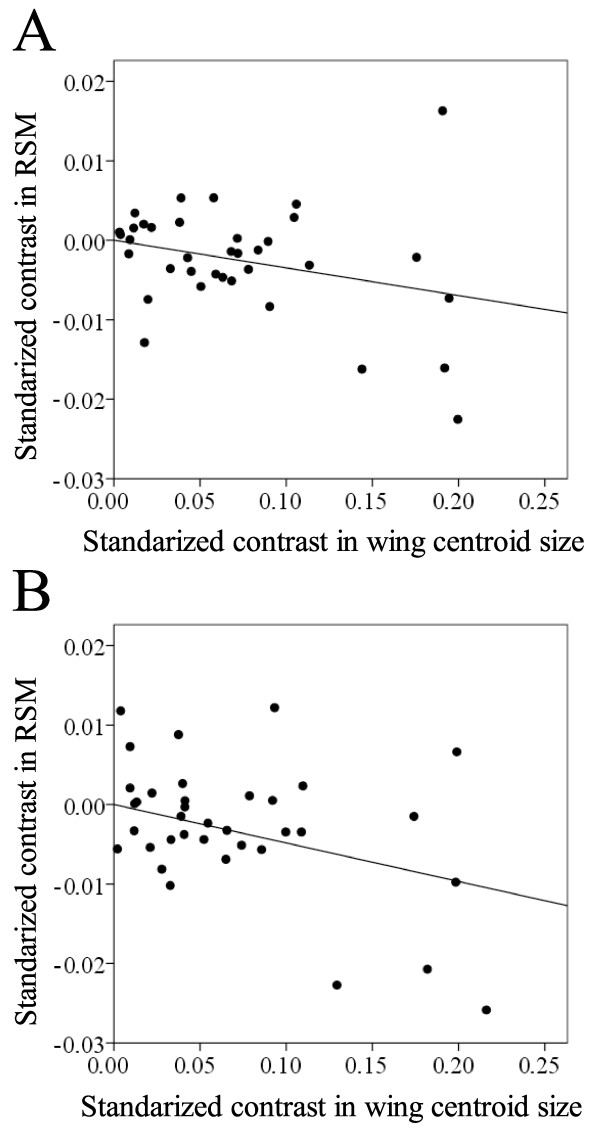
Phylogenetic independent contrasts of wing shape (measured as the non-dimensional radius of the second moment of wing area, RSM) on wing centroid size (A: forewings; B: hindwings).

After accounting for phylogenetic non-independence among our study taxa, a visual inspection of the relationship between the RSM vs. wing centroid size revealed that a quadratic term was no longer present (Figure 4). Instead, a negative linear pattern was observed (forewings: *R*^2^ = 0.194, *F*_1,35_ = 8.450, *P* = 0.006; hindwings: *R*^2^ = 0.270, *F*_1,35_ = 12.954, *P* < 0.001). Therefore, the RSM tended to decrease with wing centroid size. We also note that at smaller sizes, the values of the RSM were more constrained, while at larger sizes there was more variation (Figure 4), i.e., at large size different wing shapes were observed.

### RSM and wing coloration

In a previous study using 36 taxa and geometric morphometrics, we showed that the coloration groups differed in the non-allometric component of fore- and hindwing shape before correcting for phylogeny [28]. Moreover, the non-allometric component of hindwing shape remained significant after correcting for the phylogenetic relationships [28]. For comparative purposes, we ran a similar approach for the RSM. Before correcting for phylogeny, we did not find significant differences in the RSM among the coloration groups for forewings and marginally non-significant differences for hindwings (Table 1). A phylogenetic ANOVA on the non-allometric component of the RSM for hindwings revealed non-significant differences among the coloration groups (phylogenetic-*P* = 0.199).

**Table 1:** Results of the general linear models on the non-dimensional radius of the second moment of wing area (RSM) for fore- and hindwings

**Effect**	**d.f.**	**SS**	***F***	***P*-value**
**A. Forewings**				
Coloration group	5	0.00068	1.802	0.144
Log Centroid size	1	0.00005	0.633	0.433
(Log Centroid size)^2^	1	0.00001	0.048	0.828
Error	29	0.00220		
**B. Hindwings**				
Coloration group	5	0.00091	2.404	0.061
Log Centroid size	1	0.00085	11.279	0.002
(Log Centroid size)^2^	1	0.00003	0.351	0.558
Error	29	0.00219		

## Discussion

Our study showed a quadratic relationship between the RSM and wing centroid size, implying that small and large species have lower values of this parameter compared to intermediate-sized species. This result was somewhat surprising since a linear relationship would be expected from aerodynamic theory [15], and in a previous study we found a linear relationship between wing shape (studied with geometric morphometrics) and wing centroid size [28]. However, in the previous study [28] one of the largest species (genus *Archineura*) was not included. When we analyzed a larger size range of Odonata, we did not find the quadratic term, nor a linear relationship between the RSM and body length. These results suggest that our quadratic relationship is taxa-specific.

When phylogeny was taken into account, the RSM tended to decrease with wing centroid size. The change in this relationship after correcting for phylogenetic effects suggests a phylogenetic signal, i.e., that more closely related species tend to resemble each other more than a randomly chosen species sampled from the phylogeny. In fact, a significant phylogenetic signal of wing shape is present in this family of damselflies [28]. We also found that at small size, the values of the RSM were more constrained than at large size. All together, these results suggest that shape changes along the size axis can be only partially related to the aerodynamic demands of flight at different sizes and more comparative studies in other groups are needed to explore if this pattern is general.

The linear negative relationship between the RSM and size was a result from small species showing a higher distribution of the wing area towards the wing apex, whereas large species showed a concentration of the wing area towards the wing base. A tendency to a higher concentration of wing area towards the wing base in the large species not only promotes less-energetically demanding flight [10,47], it also results in a higher range of available flight speeds [49,50], which would be beneficial for avoiding predators and/or increasing prey captures [51-53]. The wing shape shown at smaller sizes is expected to be more energetically costly in terms of flight energetics. A possible explanation to this costly design might be the need to display during territorial behaviors. For instance, butterfly Batesian mimics were shown to pay important aerodynamic costs for flying slower and with more agility, as distasteful mimics do, improving color signaling to predators [54].

The genus *Hetaerina* had the smallest wing centroid size and was a main driver in the quadratic relationship between the RSM and wing centroid size. *Hetaerina* is the basal genus in the whole Calopterygidae family (around 150 My) [55] (see also Methods) and this is likely to be the reason why after taking into account the phylogenetic relationships the quadratic term was no longer present. Hence, *Hetaerina* is probably on a different evolutionary pathway with regard to aerodynamic design. This is supported by the fact that this genus has not evolved a courtship flight, as in more recent genera such as *Calopteryx*[29]. *Hetaerina* showed long, slender wings with a broad base and a slender tip. This shape was shared with the genus *Archineura*, which showed the largest wing centroid size and is a more recently diverged genus (less than 45 My) [55]. Interestingly, both genera share the same wing color pattern. In fact, we only found marginally non-significant differences for the RSM of hindwings (controlling for size effects) which was driven by coloration group 1 (genera *Hetaerina* and *Archineura*). However, after correcting for phylogeny, that marginal difference was no longer present. Both genera might show some level of convergence in wing shape driven by a more effective display of the wing patch at the base of the wings. In this situation, a broader wing should provide a stronger signal of the color patch [27,28]. Therefore, it is possible that the differences in the RSM among taxa are a consequence of a combination of wing size and wing coloration. However, since our results are correlative, it is still uncertain whether differences in wing shape are due to the presence of certain types of wing coloration or vice versa [28].

The lack of significant differences in the RSM of the hindwings among coloration groups after correcting for phylogeny contrasts with our previous study using geometric morphometrics, where we found significant differences [28]. Thus, our results emphasize the need for using multivariate analyses of wing shape. In fact, although we showed that the RSM was linearly related to the shape scores, we obtained quite similar values of the RSM for very different wing shapes. For example, similar values of the RSM can be gained in a short and broad wing and a long and slender wing, given that the distribution of wing area is proportionally similar between both. Therefore, although the RSM has been broadly used before and its intuitive interpretation is very useful, the use of multivariate methods such as geometric morphometrics is much more precise and should, at least, be combined with other traditional measures of wing shape.

## Conclusions

In this study we have shown that the relationships between wing shape and size are complex and taxon-specific, even within a group of species with similar flight behavior: territoriality. Moreover, the presence of wing coloration, a sexually and naturally selected trait, might influence the optimal wing shape within a trade-off between flight energetics and coloration display. The net selection pressures acting on flight performance, wing size, wing shape and body size probably differ among species, resulting in relationships that differ from the aerodynamic predictions alone. Experimental work in a comparative framework is needed to disentangle the role of wing morphology and size on flight performance.

## Methods

### Study taxa

Wing pictures of 338 males from 37 taxa of calopterygid damselflies (5–10 specimens per taxa) were collected from museum specimens (NCB Naturalis of Leiden and The Swedish Museum of Natural History, Stockholm), from colleagues or from our own samples (Table 2). Wings were either scanned in a flatbed scanner or photographed, together with a scale as a size reference. For the species of the genus *Mnais* Sélys, 1873, we used only territorial, colored morphs [56], so that only territorial taxa are included in our study.

**Table 2:** Study taxa including the sample size and the assigned coloration group (see text)

**Taxa**	**N (fore/hindwings)**	**Coloration group (fore/hindwings)**
*Archineura incarnata*	8/8	1/1
*Archineura hetaerinoides*	10/10	1/1
*Atrocalopteryx atrata*	9/10	3/3
*Caliphaea confusa*	6/6	6/6
*Calopteryx aequabilis*	10/10	4/4
*Calopteryx amata*	5/5	6/5
*Calopteryx cornelia*	10/10	2/2
*Calopteryx exul*	6/6	6/6
*Calopteryx haemorrhoidalis*	10/10	3/3
*Calopteryx maculata*	10/10	3/3
*Calopteryx splendens splendens*	10/10	4/4
*Calopteryx virgo meridionalis*	10/10	3/3
*Calopteryx virgo virgo*	10/10	3/3
*Calopteryx xanthostoma*	10/10	4/4
*Echo modesta*	10/10	6/6
*Hetaerina americana*	10/10	1/1
*Hetaerina titia*	10/10	1/1
*Matrona basilaris*	10/10	3/3
*Matronoides cyanipennis*	5/7	3/3
*Mnais andersoni*	10/10	2/2
*Mnais costalis*	10/10	2/2
*Mnais mneme*	10/10	2/2
*Mnais pruinosa*	10/10	2/2
*Mnais tenuis*	10/10	2/2
*Neurobasis chinensis*	10/10	6/3
*Phaon camerunensis*	10/10	6/6
*Phaon iridipennis*	10/10	6/6
*Phaon sp.* from Madagascar	6/6	6/6
*Psolodesmus mandarinus dorothea*	6/6	5/5
*Sapho bicolor*	10/10	4/4
*Sapho ciliata*	10/8	3/3
*Sapho gloriosa*	5/7	3/3
*Umma longistigma*	10/10	6/6
*Umma saphirina*	10/10	6/6
*Vestalis amoena*	10/10	6/6
*Vestalis gracilis*	10/10	5/5
*Vestalis lugens*	10/10	3/3

Because wing shape is at least partially associated to wing coloration [28], taxa were grouped into different categories depending on the wing coloration position, color and extension (Table 2): 1) coloration only in the wing base; 2) yellow wing coloration covering most of the wing; 3) extensive dark wing coloration covering more than 85% of the wing area; 4) less extensive dark wing coloration, covering 30-85% and located either at the central part of the wing or at the wing apex; 5) very reduced wing coloration restricted to the wing tip and covering less than 20% of the wing area; and 6) no conspicuous coloration. Notice that in some species there are differences in coloration between fore- and hindwings (Table 2).

### Wing shape analysis

**Figure 5:**
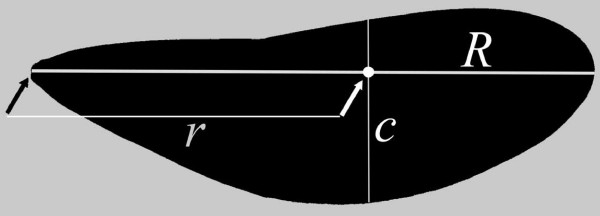
**Parameters used in the calculation of the non-dimensional radius of the second moment of wing area, RSM (see main text). ***R* represents wing length, while *r* is the radius of the wing at a certain point along the wing length and also involves a wing chord *c*.

We estimated shape using two different methods. First, we calculated the RSM. Second, we used geometric morphometrics techniques to study graphical changes in wing shape. The RSM has been used in the study of quasi-steady aerodynamics of hovering flight [10,57]. This parameter can be defined as a quantitative measure of wing area distribution along the wing axis [10] and for a pair of wings is calculated as follows:

$${\hat{r}}_{2}\left(S\right)=\int_{0}^{1}\hat{c}\hat{r}\;d\hat{r},$$

where *ĉ* is the normalized wing chord and $\hat{r}$ is the non-dimensional radius equal to *r*/*R*, where *r* is the distance to the wing base on the chord *c* and *R* is wing length (Figure 5). This parameter is proportional to the mean lift force and thus lower values involve less energetically demanding flight [10,47]. We note that the first three non-dimensional radii of moments of wing area are highly correlated [6,10]. The RSM has been used before in dragonflies and damselflies [32,50,57] and thus we decided to use this specific radius for comparative purposes. We also note that in Odonata, fore- and hindwings do not form a common functional surface as in butterflies [49,57]. Therefore, we calculated the RSM separately for fore- and for hindwings by following the protocol of Serrano-Meneses et al. [32], using ImageJ [58] and Microsoft Excel version 14.0 (Microsoft corp. 2010). We are aware that both pairs of wings may aerodynamically interact with each other, however, the aim of this study is rather descriptive and comparative.

**Figure 6:**
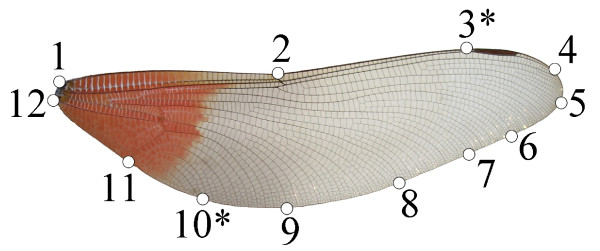
Landmarks and semi-landmarks (*) used for the study of wing shape in the geometric morphometric approach. As an example a wing of *Archineura incarnata* is shown.

We also used a geometric morphometrics approach to analyze wing shape variation. Although much more refined that the RSM, it does not provide an intuitive, quantitative estimate of wing shape as the RSM does, but it does provide an excellent visualization. In geometric morphometrics, shape is quantified from landmark coordinates after holding mathematically constant the effects of non-shape variation (position, orientation and scale) [48,59,60]. On the wing images, we digitized 12 landmarks and semi-landmarks (Figure 6) using tpsDig2 [61]. Ten biologically homologous landmarks were located at the wing base or along the wing margin where some major veins terminate. To incorporate some aspects of the wing curvature, we also used two semi-landmarks (Figure 6). Only non-damaged wings were used. The landmarks and semi-landmarks were subjected to a Generalized Procrustes Analysis (GPA), where all specimens are translated to the origin, scaled to unit centroid size, and rotated to minimize the total sums-of-squares deviations of the landmark coordinates from all specimens to the average configuration [62]. To minimize Procrustes distance between specimens during GPA, semi-landmarks were permitted to slide along their tangent directions [59,63]. A single reference shape configuration (i.e., consensus wing) was obtained. The consensus wing was used for aligning all individual shape configurations and for computing the shape components (i.e., partial warps and the uniform component) in tpsRelw [64]. Centroid size was computed for each wing and was logarithmic transformed in all analyses. Since centroid size had been previously shown to be highly correlated with body size in Calopterygidae [45,65] we used it as a proxy for body size.

### Phylogenetic tree

**Figure 7:**
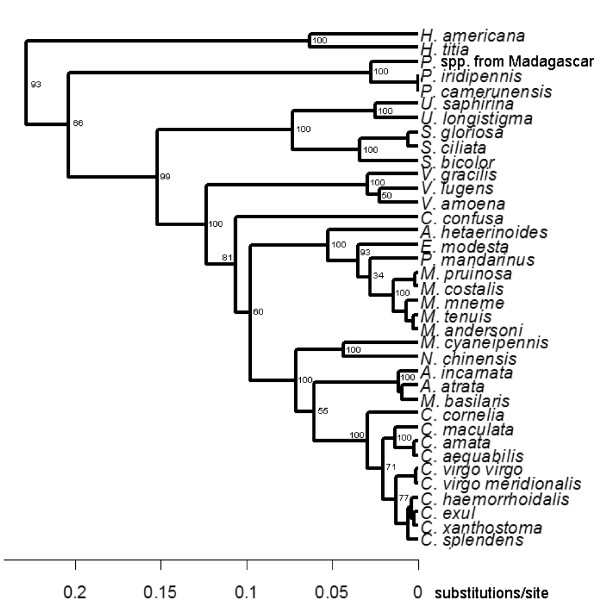
**Phylogenetic tree used in the present study (see also **[28]**).**

We used independent contrasts to correct for the phylogenetic non-independence of our study taxa. To obtain a phylogenetic tree that included all our study taxa, we re-analyzed previously published nucleotide sequences of the following genes: 18S, 5.8S, partial 28S rDNA, and of the spacers ITS1 and ITS2 [55,66-69]. We used 70 taxa of the family Calopterygidae and three more taxa as outgroups (two non-calopterygids damselflies and one dragonfly) (see Supplementary Table 1 in [28]). We aligned the sequences using the ClustalW algorithm [70] in MEGA version 5 [71]. Aligned sequences were analyzed by a Bayesian phylogenetic approach in the package BEAST version 1.7.1 [72], using a SRD06 model as the nucleotide substitution model, a relaxed molecular clock (uncorrelated lognormal) and a birth-death process as a tree prior. The Markov chain Monte Carlo sampling was run for 10^7^ generations and logged every 1,000 generations. The consensus tree was pruned and was then used for the independent contrasts (Figure 7). The tree obtained was similar to previously published trees [55].

### Statistical analyses

For all statistical tests, species were used as our level of replicate. Hence, we calculated for each species a mean value for each variable. We performed analyses both before and after accounting for the phylogenetic relationships among taxa. The statistical analyses were carried out in SPSS (IBM Corp.), unless otherwise stated.

We first performed a regression of the values of the RSM against wing centroid size. After visual inspection of the plot, we ran both a linear and a quadratic regression of the values of the RSM on wing centroid size. To visualize how wing shape was related to the variation in the values of the RSM and to wing centroid size, we computed thin-plate spline deformation grids in tpsSplin [73] using the shape components (partial warps and the uniform component). We did this by comparing the consensus wing shapes of certain taxa to the corresponding consensus wing shape, for both fore- and hindwings separately.

We also investigated how the RSM and the wing shape obtained by geometric morphometrics were related in our dataset, for fore- and hindwings separately. To do this, we first visualized this relationship by applying the approach developed by Drake and Klingenberg [74] which computes shape scores from multiple regressions of the Procrustes coordinates on a continuous variable (in our case, the RSM). The shape scores are the predicted shape variables in the regression including the residual variation in the direction of the shape space. Thus, the shape scores summarize all the landmark information into one single value. We ran this approach in R version 2.15 [75]. To statistically support the obtained relationship, we also ran a MANCOVA using the shape components from geometric morphometrics (partial warps and the uniform component) as the dependent variables. The RSM was included as a covariate and wing (fore- and hindwing) was included as a fixed factor. Since the interaction term between the RSM and wing was non-significant, it was not included in the model.

To explore how the RSM was related to size over a larger size range in Odonata, we re-analyzed data published in Serrano-Meneses et al. [32], which contains 27 taxa of non-calopterygid damselflies (smaller than our study taxa), 54 taxa of dragonflies (covering a larger size range that our study taxa) and 27 taxa of calopterygid damselflies. These data were based on the mean of the values of the RSM between fore- and hindwings and size was measured as body length. We ran an ANCOVA to compare the values of the RSM among the three groups of Odonata, with body length as the covariate.

We also studied the relationship between the RSM and wing centroid size in our dataset using phylogenetic independent contrasts applying the method described by Felsenstein [76] for examining the correlated evolution of continuous traits. We did this for fore- and hindwings separately. We used the package PDAP:PDTREE version 1.16 [77] for the Mesquite version 2.75 [78]. After inspection of the plots of the absolute values of the standardized phylogenetically independent contrasts versus their standard deviations, we transformed the branch lengths of the tree to satisfy the requirements of the independent contrasts [79]. All the obtained results were equivalent for both wings, so we only show the results for the exponentially transformed branch lengths. The plots of contrasts were standardized for both axes and also positivized for the X axis [79]. After visual inspection of the relationships, least squared regressions were performed on the contrasts.

Finally, we inspected whether there were differences among the coloration groups in the values of the RSM. We have previously shown similar results for a dataset of 36 taxa of the same family, but using geometric morphometrics approaches [28]. In the present study, we asked a similar question for comparative purposes between the two methods of measuring wing shape. We first explored the effect of wing coloration on wing shape without taking into account the phylogenetic effects. We ran a general linear model including the RSM as the response variable, wing centroid size and squared wing centroid size as covariates, and coloration group as a fixed factor, separately for fore- and hindwings. Non-significant interactions were removed one by one from the model. Second, we explored the effect of wing coloration on the RSM controlling for the phylogenetic non-independence. We removed the size effects on the RSM by performing a regression for each wing (fore- and hindwings). To correct for the quadratic relationship between the RSM and wing centroid size, we included in the model wing centroid size and squared wing centroid size as covariates. We saved the residuals from the regression, i.e., the non-allometric component of the RSM. We used this size-corrected RSM for examining the differences among the coloration groups from a phylogenetic perspective by running a phylogenetic ANOVA [80], for both fore- and hindwings separately. In this case, we used the R-package geiger [81], which returns a *P*-value based on Brownian motion simulations.

## Competing interests

The authors declare that they have no competing interests.

## Authors’ contributions

DO coordinated sample collection, did data acquisition, analyzed data, interpreted the results and wrote the manuscript. DCA analyzed data, interpreted the results and revised the manuscript. FJ coordinated sample collection, interpreted the results and co-wrote the manuscript. All authors read and approved this paper.
